# Surgery

**Published:** 1873-06

**Authors:** 


### ARTICLE VIII.

### *Surgery.*

On the Removal of Tumors from Bone—The object of this short but valuable paper (Sir James Paget in the *Medico-Chirurgical Transaction*, Vol. L I V, London, 1871,) is to formularize and recommend a mode of treatment, which though it has been occasionally resorted to in individual cases, has never been recognized as an established rule of practice. The mode of treatment in question is the enucleation, or simple extirpation, of nonmalignant tumors growing in bone, rather than their removal by the more sweeping operations of resection or amputation. Six cases are detailed to show the feasibility and advantage of thus dealing with cartilaginous, bony, fibrous and myeloid tumors growing in bone; and the paper ends with certain suggestions as to diagnosis, which appear to us so valuable that we quote them in full for the benefit of our readers:

“For cancerous and recurrent tumors, amputation or resection is generally advisable; for innocent tumors growing on bones, excision; for innocent tumors growing in bones, enucleation.

"1. The tumor is probably cancerous if its growth commenced before puberty, or after middle age, unless it be a cartilaginous or bony tumor on a finger or toe, or near an articulation.

"2. If a tumor has existed on or in a bone for two or more years, and is still of doubtful nature, it is probably not cancerous or recurrent, and this probability increases with the increasing duration of the tumor.

"3. If a tumor on or in a bone has doubled or more than doubled its size in six months, and is not inflamed, it is probably cancerous or recurrent, and this probability is increased if among the usual coincidences of rapid growth, the veins over the tumor have much enlarged, or the tumor have protruded far through ulcerated openings, and bleeds and profusely discharges ichor.

"4. If with any such tumor, not being inflamed, the lymphatics near it are enlarged, is probably cancerous, and still more probably if the patient have lost weight and strength to amounts more porportionate to the damage of health by pain, or fever, or other accident of the tumor.

"5. A tumor on the shaft of any bone but a phalanx is rarely innocent, and so are any but cartilaginous outgrowths on the pelvis, or any but the hard, bony tumors on the bones of the skull.

"When the wall of the bone can be traced over the surface, or any part of the surface, or the tumor, its growth from within is almost certain. And so it is when, on the surface of the tumor, portions of bone can be felt among portions of more yielding substance. General smoothness of surface is usually significant of a tumor growing with a bone and expanding it, unless in the case of cartilginous tumors, which, after growing within bones, have been protruded through some parts of their expanded walls. Pulsation in a non-cancerous tumor connected with bone is a nearly certain sign of growth within bone, except in the cases of certain specimens of myeloid epulis ; and, when such pulsation is felt, it is no indication that severe bleeding will ensue in the removal of the tumor, for it is only derived from the arteries on the walls of the bone cavity lodging the tumor. "—*American Journal Medical Sciences.*

